# Non-contributory pension programs and frailty of older adults: Evidence from Mexico

**DOI:** 10.1371/journal.pone.0206792

**Published:** 2018-11-02

**Authors:** Emma Aguila, Mariana López-Ortega, Luis Miguel Gutiérrez Robledo

**Affiliations:** 1 Sol Price School of Public Policy, University of Southern California, Los Angeles, CA, United States of America; 2 RAND, Santa Monica, CA, United States of America; 3 Research Department, National Institute of Geriatrics, National Institutes of Health, Mexico City, Mexico; 4 Office of the Director General, National Institute of Geriatrics, National Institutes of Health, Mexico City, Mexico; University of East Anglia, UNITED KINGDOM

## Abstract

Non-contributory pension programs in the developing world seek to provide older adults with an income that may improve their health and wellbeing in old age by enabling access to health care and better nutrition. There is no previous evidence of the effects of non-contributory pensions on frailty, a comprehensive measure of health and well-being of the oldest old. We aimed to estimate the effects of non-contributory pension programs on frailty of older adults in the state of Yucatan, Mexico. We use rich panel data, including objective markers and self-reported assessments of health and well-being, for 944 adults at least 70 years of age in two communities of Yucatan, Mexico. The first wave was collected in 2008; the second wave was collected in 2010, 18 months after implementation of a monthly state pension in one community and 12 months after a federal pension paid every two months in the other. We found the state pension led to a statistically significant decrease in the severity of frailty for women, but the federal pension was associated with an increase. We found no statistically significant change in the frailty index for men in either community. Among explanations for these findings are monthly payments being more likely to be spent on health care, medicines, and more regular food expenditures, enabling women who previously lacked independent means of support to increase their longer-term health. The federal program paid every two months led to irregular patterns of food expenditure and increased ownership of durable goods but had no effects on health care utilization, subsequently leading to deterioration in longer-term health for women.

## Introduction

Mexico is aging rapidly. From 2010 to 2050, the population 60 years and older is projected to increase from 8.9% to 21.5% of the total population [1]. This growth will occur within a “mixed” epidemiological transition where increasing prevalence of chronic diseases coexists with continuing moderate or high incidence of infectious diseases [2]. Such growth reflects that occurring elsewhere; globally, older populations are increasing more rapidly than any other age group.

Poverty in old age is common in middle- and low-income countries [3]. In Mexico in 2014, 21.2% of adults 65 years or older had a monthly income below the minimum welfare line of US$131.40 in purchasing power parity (PPP) [4,5]. Poverty in old age in Mexico is partly due to the lack of universal coverage of the social security system. Mexico, like other middle- and low- income countries, has limited support for an aging population, leaving families primarily in charge of providing care and economic security for older adults [2].

Mexican workers contribute to a three-party (worker, employer, and government) public social security system and receive social security benefits when they retire. Most workers—52.1%—are, however, in the informal sector and do not contribute to social security [6]. Informal sector workers tend to be self-employed in low-wage jobs or in noncompliant private firms that do not offer workers the opportunity to participate in government social security programs [7]. Social-security and health-care services contributions are not mandatory for self-employed workers, leading such workers to skip contributions to the detriment of receiving health insurance and eligibility for social-security benefits at retirement. As a result, in 2016, only 31.0% of Mexican workers received social security benefits [8].

In many low- and middle-income countries, governments have introduced safety net programs that provide cash and other benefits such as a basic food basket, free meals, and subsidies to help pay for utilities, health insurance, or health care [9–12]. For example, in Jamaica poor older adults receive free medical care and a cash transfer [12]. The United States provides food price subsidies, such as food stamp programs, and in-kind food assistance programs that have been extensively analyzed [13]. Other well-known conditional cash transfer programs have been introduced in Brazil and Mexico. In Mexico, cash and food assistance transfers for older adults living in eligible low-income households conditional on attending nutrition and health training sessions, were implemented with the expansion of a program targeted to children [14].

Unconditional cash-transfer schemes or non-contributory pensions for older adults are the main means for providing income security in old age in at least 40 countries around the world including Argentina, Brazil, Bangladesh, Mexico, and South Africa [9, 15]. The Mexican federal government introduced a non-contributory pension program for adults 70 years or older in rural locations with less than 2,500 inhabitants in 2007, expanding it to all localities with fewer than 20,000 inhabitants in 2008, to all localities with fewer than 30,000 inhabitants in 2009 when it was renamed *Programa 70 y Más* (70 y Más), to all older persons not receiving any other social security benefits or state-level non-contributory pensions in 2012, and to all such persons at least 65 years of age in 2013, when the program was renamed *Programa Pensión para Adultos Mayores* [16]. Prior to the introduction of the federal program, many states introduced their own non-contributory pensions: Mexico City in 2001, Chihuahua and Nuevo Leon in 2004, Quintana Roo in 2006, and Chiapas, Sinaloa, and Yucatan in 2007. Currently, most older adults in Mexico rely on state or federal non-contributory pension benefits, which are significantly smaller than social security benefits [7].

Previous research has found mixed effects of non-contributory pensions on social and health outcomes. In Mexico, the federal government non-contributory pension program (NCP) that disburses pension payments every two months, has reduced poverty [17], improved mental health [18], reduced depressive symptoms, increased participation and empowerment in household spending decision making for older recipients [19], and increased household consumption [18]. In Yucatan, a monthly state-level NCP has improved verbal recall and lung function, reduced anemia, and increased use of health-care services and affordability to buy medicines [20]. Relative to the federal pension paid every two months, the state pension paid every month in Yucatan was more likely to boost health care utilization and food availability [21]. At the same time, both federal and state NCP in Mexico have reduced private family transfers to older adults [20,22,23], reduced saving rate of households [24], and reduced paid work by recipients [18,20,25].

In Brazil, NCP have reduced poverty [26–29] and household vulnerability [29], improved access to health services and medicines [27], and improved subjective wellbeing and optimism about the future among recipients [30]. At the same time, they reduced labor force participation among recipients [31]. A comparative study of Argentina, Colombia, Costa Rica, Chile, Dominican Republic, El Salvador, Honduras, Panama, Paraguay, Peru, and Uruguay found NCP reduced poverty [17]. In South Africa, some research has indicated that NCP have reduced household poverty, inequality [29,32], and household vulnerability [29]; increased local credit; smoothed consumption[33]; improved self-reported health [34]; and led to greater health-care use and hypertension awareness[35].At the same time, research for the South African case has found no effect of on control of hypertension or on self-reported health and quality of life [35].

Altogether, research across several countries indicates NCP can reduce poverty but have less conclusive effects on health [35]. Among possible reasons for the less conclusive research are the limitations of previously collected health data and the difficulties in measuring the relationship between income and health. Regarding the limitations of previously collected health data, we note that much research has relied on self-reported health and other subjective measures of wellbeing. While such measures are easier and less costly to collect than more objective ones, they may not always be accurate predictors of health status [36].

In recent decades, several indicators of health and well-being of older adults have been developed to better describe the heterogeneity of this population and its chronic conditions, functional disability, sensory impairment, and other ailments. The first comprehensive assessments focused on individual ability to function independently as measured by the ability to perform daily and instrumental activities of daily living [37,38] including personal care, toileting, preparing meals, feeding, shopping, and managing money [39]. More recently, the concept of Frailty, defined as the result of decreased homeostatic reserve and resistance to stress [40,41], was developed to describe the condition associated with increased risk of functional decline among older adults. Frailty is now one of the most-used indicators of health and physical well-being in older adults. It has been studied extensively in high-, middle-, and lower- income countries, and has been shown to be a strong predictor of adverse outcomes such as falls, hospitalizations, and death [42,43]. Previous studies for Mexico confirm that frailty is more prevalent in women and is associated with low socioeconomic status [44–46]. To our knowledge, no studies have explored the effect of non-contributory pensions on frailty or other comprehensive and objective measures of health and wellbeing for older adults.

Regarding the difficulties in measuring the relationship between income and health, we note establishing causality here is notoriously difficult because it can run both ways (e.g., Smith [47]). That is, while income may affect health, health may also affect income. Our work, through its use of experimental data, provides a unique opportunity to disentangle longer-term effects between income and health.

We analyze the effect of NCP on frailty, a more-comprehensive measure of health and functional ability using biomarkers. We use rich panel data collected in the state of Yucatan, Mexico, including objective markers and self-reported questions on health and wellbeing, collected before (August-November 2008) and after (June-August 2010) the introduction of two NCP, one state and one federal. The communities where these programs were introduced are similar in their location, socioeconomic and demographic characteristics, and pensions that older adults received. We therefore expect the introduction of the pension programs to have similar effects. We attribute differences in the outcome variables to the state program paying its benefit more frequently than the federal one.

In addition to increasing purchasing power, NCP income can enable better food availability and purchase and use of medicines and health services, including education, nutrition, and other care programs. We hypothesize that, by reducing financial constraints, the NCP improved food intake and use of health care services, with these in turn having longer-term effects on nutrition, health, and wellbeing, ultimately stabilizing or even reducing the level of frailty for recipients.

## Methods

### Study design and participants

Our research focuses on the effects of two NCP programs: *Reconocer Urbano*, a state-level program in Yucatan, and *70 y Más* (as it was named during the period of analysis for this study), a federal program across Mexico. *Reconocer Urbano* was rolled out in stages with an experimental design to evaluate its impact on the health and well-being of older adults by town. The experimental design included two communities with similar socioeconomic and demographic characteristics and geographic location in the northeastern part of the state: Valladolid (45,868 inhabitants) and Motul (21,508 inhabitants).

The introduction of the NCP in the state of Yucatan was done in three phases. The first phase included localities with less than 6,500 inhabitants; the second phase was for localities with less than 20,000 inhabitants; and the third phase was for localities with 20,000 inhabitants or more. Eleven cities in the state qualified for the third phase of the program (Hunucma, Kanasin, Uman, Merida, Motul, Oxcutzcab, Progreso, Tekax, Ticul, Tizimin, and Valladolid). From these cities, we conducted a cluster randomized controlled trial design by matched pairs of towns with similar characteristics using the 2005 Mexican Census. Among those pairs, one was randomly chosen, Valladolid and Motul; within that pair, Valladolid was randomly chosen to start the state program non-contributory pension in 2008.

As shown in S1 Table, both towns are similar in their poverty index, proportion of illiterate population, and household characteristics. We also conducted a community survey to understand in more detail the differences at baseline and through time between the two cities in their healthcare infrastructure, economic activity, and government programs. The economic activity in both towns includes manufacturing (textile, automotive, wood, plastic, etc.), assembly plants, construction, wholesale and retail commerce, restaurants, and hotels. In addition, Valladolid has some agricultural employment. Both towns had similar federal government programs and state government programs (see S2 Table) except for NCP programs. Both towns also have clinics of the Mexican Social Security Institute (IMSS), and of the Ministry of Health of the Government of the State of Yucatan. Motul has fewer hospitals than Valladolid but Valladolid has more inhabitants. We did not find changes in the infrastructure or economic activity nor did we find differences in natural disasters or air quality in the analysis period. S1 Fig. and S3 Table show both towns followed similar socioeconomic trends over time.

We identified participants for the pension program by screening all households with adults 70 or older. We did so in partnership with the National Institute of Statistics in Mexico (INEGI). INEGI provided us with maps of the evaluation communities, updated the maps as necessary (with a cartographer accompanying our data-collection team), helped train the data-collection team, and provided quality assurance during the listing process. In screening the selected communities, interviewers listed each household to identify age-eligible respondents and, using a brief form, collected first and last names, age, date of birth, gender, and preferred interview language (Spanish or Mayan). A detailed description of the study design, sampling frame, and follow-up procedures has been previously published [48].

Valladolid started receiving a state NCP of MXN$550 (US$58.70 per month at 2014 purchasing power parity, or PPP) in December 2008, paid monthly to persons 70 and older. Prior to this, we conducted baseline surveys (W1) between August and November 2008 among all households with persons 70 or older in Valladolid and Motul. In July 2009, the federal government expanded its program *70 y Más*, and households with adults 70 years and older in Motul, but not in Valladolid, became eligible for the federal NCP of MXN$1,000 (or US$105.60 at 2014 PPP), paid every two months. In short, both the state and federal programs provide a very similar benefit but at different frequency: monthly for the state program and every two months for the federal program.

We conducted a follow-up survey (W2) in June and July 2010, 18 months after Valladolid began receiving *Reconocer Urbano* and 12 months after Motul started receiving *70 y Más*. We calculated response rates using American Association for Public Opinion Research guidelines [49]. Response rates at W1 were 94.3% in Valladolid and 96.7% in Motul; at W2, they were 85.9% in Valladolid and 85.6% in Motul. In September 2012, *Reconocer Urbano* was replaced by the program *Reconocer Universal*, and rolled out to all older adults in Yucatan.

The survey questionnaires we used were comparable to those for the Mexican Health and Aging Study (MHAS) and the U.S. Health and Retirement Study (HRS), and included a comprehensive assessment of health, disability, and socioeconomic characteristics. The surveys also collected anthropometric measurements (height, weight, waist circumference) and performance measures (lung capacity, gait speed, grip strength) for age-eligible respondents. We built a team of bilingual (Spanish and Mayan, the local indigenous language) data collectors for this research project. We provided the interviewers more than 250 hours of training in general interviewing techniques, question specifications, refusal, informed consent, the use of proxy respondents and secondary informants, guidelines for handling common problems, data confidentiality and safeguarding, field safety, issues in surveying identified respondents, and protocols for managing case assignments. We also trained and certified all data collectors in collecting anthropometric measurements and biomarkers following the standardized protocols and equipment of HRS [50]. We conducted training through classroom lectures, practicing in pairs using computers, and pretesting under actual field conditions. To evaluate interviewer performance and adherence to study protocols, all staff received additional training on the measurement of biomarkers (including formal assessments) approximately every six months. We conducted the survey using CAPI (Computer Assisted Personal Interviewing).

The original sample consisted of 1,186 men and 1,265 women in both communities. Of those, 1,136 men and 1,215 women responded to the W1 survey, with the remaining 4.1% of the sample not interviewed because of refusal or changed address since the initial listing. Of those responding to the W1 survey, 793 men and 854 women responded to the W2 survey as well, with the remaining 29.9% not interviewed at W2 because of death, refusal or changed address. After dropping cases with missing information for our frailty index (discussed below), we had a final working sample of 470 men and 474 women.

The Internal Review Board at RAND Corporation revised and approved the protocol (approval number 2008-0513-CR07) for the surveys. The study complied with U.S. and Mexican requirements and standards for conducting ethical research. An informed-consent form that followed the Helsinki Declaration II was provided to each participant. Informed written consent was obtained separately for anthropometric and biomarker assessments. We obtained oral consent for the general interview, while for anthropometric and biomarker measurements we obtained written informed consent. Participants who did not provide their consent in either were not interviewed [51].

### Outcomes

#### Frailty

We generated a frailty index using a slight modification of Fried’s frailty phenotype [40], including the following survey components.

#### Weight loss

Respondents classified with weight loss were those reporting they had unintentional loss of at least 3 kg in the three months before W2 or whose measured weight loss between W1 and W2 was at least 3 kg and reported as unintentional.

#### Grip strength (weakness)

We measured grip strength in kilograms using a hand-held Smedley’s brand dynamometer on the respondent’s dominant hand. A trained interviewer administered the test and took two measurements for each participant. Classifying men and women separately, we designated as weak and gave a score of 1 to respondents who were unable to perform the test or who were in the lowest quintile in grip strength for each body-mass index; quintile defined at baseline.

#### Exhaustion (poor endurance and energy)

We identified self-reported exhaustion with a question from the Composite International Diagnostic Interview-Short Form (CIDI-SF) test that evaluates major depressive episodes as defined by the revised third edition of the *Diagnostic and Statistical Manual of Mental Disorders of the American Psychiatric* Association [52]. Participants who responded “Yes” to the question “Did you feel more tired out or did you have less energy than is usual for you?” were scored as 1 for this criterion.

#### Walking speed (slowness)

We assessed walking speed with a timed 12-feet-long walk test. Interviewers placed measuring tape for the walk and made sure participants were wearing appropriate footwear before asking them to complete the walk at their usual pace. The test was completed two times and the faster time recorded. Classifying men and women separately, we coded as 1 those unable to perform the test or who were in the lowest quintile in walking time for those of similar height group (i.e., either no more than mean height or above mean height) fixed at baseline.

#### Physical activity

We assessed self-reported physical activity using the question “On average during the last month, have you exercised or done hard physical work three or more times a week?” This question includes sports, heavy household chores, or other physical work. We scored as 1 those who responded “No” to this question.

After generating these criteria, we constructed a summary measure of deficits (0–5). Following Fried and colleagues [40], we classified those who scored 0 on the summary measure as not frail, those who scored 1 or 2 as pre-frail, and those who scored 3 or higher as frail.

### Covariates

Our covariates include age, age squared, marital status (1 = married or consensual union, 0 = otherwise), total years of formal education, number of household members, whether respondent lives alone (1 = yes, 0 = no), study wave (1 = W2, 0 = W1), whether respondent lives in Vallodolid and received the state pension (1) or Motul and received the federal pension (0), and an interaction variable between W2 and the indicator for the state program (1 = state program * W2, 0 = otherwise). We also included other baseline covariates that could affect the risk of frailty. These include number of chronic conditions, health insurance (1 = yes, 0 = no), health care utilization, nutrition, and a local poverty index. Chronic conditions include cancer, hypertension, diabetes, respiratory problems, liver disease, stroke, and heart disease. Health care utilization is the number of doctor visits in the last three months. Nutrition includes questions: “How often do you eat meat, poultry or fish”, “How often do you eat fruits or vegetables”, “How often do you eat tortilla, bread, crackers or other cereals”, each with a four-point scale ranging from never (1) to at least once per day (4). The poverty index, a continuous variable with values from -1.098 to 1.409, where higher values indicate higher poverty levels, was computed by the Mexican National Population Council (CONAPO) at the level of the AGEB, a basic geo-statistical area of a group of blocks within a geographic area [53].

### Statistical analysis

We calculated descriptive statistics of outcomes and estimated the impact of the non-contributory pension program by comparing the differences between the towns receiving state and federal programs using differences-in-mean outcomes between W1 and W2 (pre vs post estimator). We compare the change in outcomes for the two programs (difference-in-differences or DID estimator) to identify the causal effect of the state program with respect to the federal program, assuming both towns follow common trends or similar natural tendency in the absence of treatment. The common-trends assumption allows the confounded time trend of the pre-versus-post estimators to drop out when conducting the DID analysis that identifies the causal effect. Differences in levels between towns are corrected by the DID estimator but both towns must follow similar trends before the introduction of the NCP [54].

Both towns had similar characteristics at baseline and had similar economic trends (see S1 Fig. and S1 and S3 Tables). Previous analysis of this field experiment [21] found both towns satisfy the common-trends assumption. Also, both towns had similar access to federal and state programs. S2 Table shows that the state- and federal-program towns had a relatively low coverage of *Oportunidades*, an unconditional cash transfer program for households in poverty (11.1% in the state-program town and 17.7% in the federal-program town). There were no programs besides the NCP implemented differentially for the two towns.

To test multiple hypotheses, we apply a Holm-Bonferroni correction [55]. In this analysis, we identify the effects of the state program 18 months after its introduction with respect to the effects of federal program 12 months after its introduction. We excluded from analysis participants missing more than 20% of data for variables in the frailty index, resulting in dropping 20.2% of the sample (17.2% of the men and 22.9% of the women). To test robustness of the results, we use parametric OLS regression with covariates not affected by the program. We conducted the following intention-to-treat (ITT) differences-in-difference (DID) regression:

$$Y_{it}=\beta_{0}+\beta_{1}w_{t}+\beta_{2}P_{i}+\beta_{3}\left(P_{i}*w_{t}\right)+\beta_{4}X_{it}+\varepsilon_{it}$$ (1)

where *Y*_*it*_ is the outcome of interest for individual *i* in wave *t*, *w*_*t*_ is a dummy variable for wave (W2 = 1, W1 = 0), *P* is a program dummy (state = 1, federal = 0), and *X*_*it*_ includes demographic characteristics and health related outcomes that could affect the risk of frailty. The parameter *β*_3_ measures the difference in the treatment effects between W1 and W2 for the state and federal programs. The role of covariates is to improve the precision of the difference-in-difference estimate *β*_3_. We estimate robust and clustered standard errors at the household level.

The estimated coefficient of the interaction between W2 and the indicator for Valladolid, the state-program town, shows the regression equivalent of the mean differences in the changes between the groups’ results. We also use a non-parametric statistical procedure, propensity score matching (PSM), to compare individuals with similar characteristics in the state- and federal-program towns, and use the same set of variables for PSM that were used in the OLS regressions. We estimated propensity scores using a probit model. S2 Fig. shows the density curves of the propensity scores before matching. We impose region of common support and drop very few observations. We compute Kernel matching and use the Epanechnikov kernel function. We compute standard errors using the bootstrap method with 1,000 replications. Propensity-score matching has a more adaptable weighting system than OLS to balance the characteristics between groups [54].

To assess potential sample selection issues, we conducted four robustness analyses: (a) we compared W1 characteristics of individuals who died prior to W2 with those who remained alive to assess the effects of mortality bias; (b) we compared W1 characteristics of individuals who did not complete W2 with longitudinal respondents to assess sample attrition bias; (c) we compared characteristics (age and gender collected in the listing) of the not-completed and completed interviews between the listing and W1; (d) we analyzed item non-response per variable in the frailty index to assess the quality of the data and other potential bias.

## Results

The final working sample at baseline consisted of 528 adults 70 years and older (54.3% women) in Valladolid, the state-program town, and 416 (44.9% women) in Motul, the federal-program town. Table 1 presents the descriptive statistics of the sample. The mean age of respondents at baseline was 76.8 years (±5.5SD) for men and 75.5 years (±.5.0SD) for women in the state-program town and 75.8 years (± 5.2SD) for men and 75.0 years for women (±5.7SD) in the federal program town. In the state-program town, 22.6% of men and 58.6% of women were single (including widowed, divorced or separated) at baseline survey; in the federal-program town, 33.3% of men and 48.9% of women were single. These differences between towns were statistically significant for married and single men and women (p<0.05). Educational attainment is low in both towns for both men and women, at an average of approximately 2 years of schooling (±2.1SD). Mean number of household residents was also similar in both towns and for both men and women, with around 3.5 persons (±2.1SD) per household. Between 11% and 15% of respondents reported living alone at the time of baseline survey, with women slightly more likely to do so.

**Table 1:** Descriptive statistics.

	State Program (Valladolid) % or Mean (SD)	Federal Program (Motul) % or Mean (SD)	Difference	*P*	State Program (Valladolid) % or Mean (SD)	Federal Program (Motul) % or Mean (SD)	Difference	*P*
	Men				Women			
*Covariates*								
Age	76.7 (5.5)	75.8 (5.2)	-1.0	0.049	75.5 (5.0)	75.0 (5.7)	-0.5	0.305
Marital status								
Single/Divorced/Separated/Widowed	22.6	33.3	10.7	0.010	58.5	48.9	-9.6	0.041
Married or consensual union	77.4	66.7	-10.7	0.010	41.5	51.1	9.6	0.041
Mean years of education	2.5 (2.9)	2.2 (1.7)	-0.2	0.260	1.9 (2.4)	2 (2)	0.2	0.419
Live alone	11.5	11.8	0.3	0.914	11.5	15.0	3.5	0.282
Mean no. of household residents	3.3 (2.1)	3.7 (2.2)	0.3	0.095	3.4 (2.1)	3.5 (2.1)	0.1	0.632
Number of chronic conditions	0.5 (0.7)	0.4 (0.6)	-0.1	0.276	0.6 (0.8)	0.7 (0.9)	0.1	0.369
Health insurance [1 = yes, 0 = no]	73.4	76.7	3.2	0.422	70.8	78.7	7.9	0.052
Number of doctor visits	1 (1.5)	1 (1.5)	0.0	0.815	1.1 (2)	1.2 (1.5)	0.1	0.522
Nutrition [1 = Never—4 = Once a day]								
Meat, poultry or fish	2.8 (0.7)	2.8 (0.6)	0.0	0.980	2.8 (0.7)	2.8 (0.6)	0.0	0.461
Fruits or vegetables	2.8 (0.8)	2.8 (0.8)	0.0	0.717	2.9 (0.8)	2.9 (0.8)	0.0	0.628
Tortillas, bread, or crackers	3.9 (0.3)	4 (0.2)	0.0	0.142	3.9 (0.3)	3.9 (0.3)	0.0	0.490
Poverty Index	0.1 (0.5)	0.4 (0.5)	0.3	0.000	0.1 (0.4)	0.4 (0.5)	0.3	0.000
*Outcomes*								
Weight loss	0.1 (0.3)	0.1 (0.3)	0.0	0.428	0.1 (0.3)	0.1 (0.3)	0.0	0.453
Weakness	18.3	21.9	3.6	0.340	19.6	16.3	-3.4	0.368
Exhaustion	11.4	6.0	-5.3	0.047	16.7	10.3	-6.3	0.056
Slow pace	19.5	16.7	-2.8	0.453	17.3	14.5	-2.8	0.432
Low physical activity	58.4	53.9	-4.5	0.328	62.4	65.8	3.4	0.450
Frailty Index [0 to 5]	116.9	103.9	-12.9	0.138	124.7	111.8	-13.0	0.123
Frailty Level	0.8 (0.6)	0.8 (0.6)	-0.1	0.299	0.9 (0.6)	0.8 (0.5)	-0.1	0.201
Not frail [0]	25.9	29.4	3.5	0.403	21.6	23.0	1.4	0.723
Pre-frail [1]	64.6	63.2	-1.5	0.744	66.9	70.6	3.7	0.397
Frail [2]	9.5	7.5	-2.0	0.434	11.5	6.4	-5.1	0.052
No. Observations	243	228			287	187		

Notes: SD = Standard deviation

Respondents reported an average of 0.5 (±0.7SD) chronic conditions each, and 70% to 79% reported access to health insurance. On average, respondents report one doctor visit (±1.5SD) in the previous three months. Men and women report similar frequency of consumption for meat, poultry or fish, (2.8 or at least once a week with ±0.7SD), for fruits or vegetables (2.8 or at least once a week with ±0.8SD), and for tortilla, bread, or crackers (3.9 or at least once a day with ±0.3SD). The average AGEB-level poverty index is higher (0.4 with ±0.5SD) in Motul, the federal-program town, than in Valladolid, the state-program town (0.1 with ±0.5SD), and the differences are statistically significant. Nevertheless, as mentioned in the methods section, differences in levels are corrected by the DID estimator and do not bias results of our analysis. There were differences between Valladolid and Motul in most of our frailty measures, but these differences were not statistically significant. As expected, we observe substantial differences between men and women in the level of frailty. In Valladolid 21.6% of women and 25.9% of men were not frail at baseline; in Motul, 23.0% of women and 29.4% of men were not frail (Table 1).

Table 2 shows that the frailty index decreased for women and men in Valladolid, the state-program town, between W1 and W2, 18 months after the intervention. The effects are larger and statistically significant for women, for whom the five-point frailty index decreased by 0.27, from 1.25 in W1. For men in Valladolid, the frailty index decreased by 0.06 from 1.17 in W1. In Motul, the federal-program town, the frailty index for women showed a statistically significant increase of 0.16 from 1.11 in W1, while that for men increased by 0.08 from 1.04, an amount that was not statistically significant. The decrease in frailty for Valladolid appears to be a result of a decrease in exhaustion and in the proportion of women with a slow pace. The increase in frailty for Motul appears to be a result of an increase in weakness for women.

**Table 2:** Effects of the Noncontributory Pension Programs on Frailty (W1, W2).

	State Program (Valladolid) W1	Federal Program (Motul) W1	State Program (Valladolid) W2-W1		Federal Program (Motul) W2-W1		State-Federal W2-W1			
	Mean (SE)	Mean (SE)	Diff. (SE)	*P*	Diff. (SE)	*P*	Diff. (SE)	95% CI	*P*	HB
Men										
Weight loss	0.10 (0.02)	0.08 (0.02)	0.02 (0.02)	0.270	0.03 (0.02)	0.146	-0.01 (0.03)	(-0.06, 0.05)	0.831	
Weakness	0.18 (0.03)	0.22 (0.03)	0.06 (0.02)	0.008	0.06 (0.02)	0.008	0.00 (0.03)	(-0.06, 0.06)	0.915	
Exhaustion	0.11 (0.02)	0.06 (0.02)	-0.09 (0.02)	0.000	-0.02 (0.01)	0.086	-0.06 (0.02)	(-0.11, -0.02)	0.002	††
Slow pace	0.18 (0.03)	0.15 (0.03)	-0.01 (0.03)	0.684	-0.03 (0.02)	0.205	0.02 (0.03)	(-0.05, 0.08)	0.626	
Low physical activity	0.58 (0.03)	0.54 (0.03)	-0.02 (0.03)	0.459	0.05 (0.03)	0.093	-0.07 (0.04)	(-0.16, 0.01)	0.080	
Frailty Index [0 to 5]	1.17 (0.06)	1.04 (0.06)	-0.06 (0.05)	0.241	0.08 (0.05)	0.124	-0.14 (0.07)	(-0.28, 0.00)	0.056	
Frailty Level [0 = Not frail, 1 = pre-frail, and 2 = frail]	0.84 (0.04)	0.78 (0.04)	-0.05 (0.03)	0.100	0.00 (0.03)	0.892	-0.05 (0.05)	(-0.14, 0.04)	0.285	
No. Observations	243	228								
Women										
Weight loss	0.10 (0.02)	0.08 (0.02)	0.00 (0.02)	0.821	0.04 (0.02)	0.109	-0.04 (0.03)	(-0.09, 0.02)	0.157	
Weakness	0.20 (0.03)	0.15 (0.03)	0.00 (0.02)	0.840	0.15 (0.03)	0.000	-0.16 (0.04)	(-0.23, -0.09)	0.000	††
Exhaustion	0.16 (0.02)	0.11 (0.02)	-0.11 (0.02)	0.000	-0.01 (0.02)	0.528	-0.10 (0.03)	(-0.15, -0.05)	0.000	††
Slow pace	0.17 (0.02)	0.14 (0.03)	-0.08 (0.02)	0.000	0.01 (0.02)	0.793	-0.08 (0.03)	(-0.15, -0.02)	0.008	††
Low physical activity	0.62 (0.03)	0.66 (0.03)	-0.06 (0.03)	0.047	0.00 (0.03)	1.000	-0.06 (0.04)	(-0.14, 0.03)	0.195	
Frailty Index [0 to 5]	1.25 (0.06)	1.12 (0.06)	-0.27 (0.05)	0.000	0.16 (0.06)	0.004	-0.43 (0.07)	(-0.57, -0.29)	0.000	††
Frailty Level [0 = Not frail, 1 = pre-frail, and 2 = frail]	0.90 (0.03)	0.83 (0.04)	-0.15 (0.03)	0.000	0.07 (0.03)	0.036	-0.22 (0.04)	(-0.31, -0.13)	0.000	††
No. Observations	286	187								

Notes: Diff. = Difference; SE = Standard errors

††p < .05 after HB correction.

Overall, the frailty index showed an important improvement for women receiving the state pension. In comparison to those who received the federal program, women who received the state program decreased their frailty severity by -0.43 (95% CI -0.57, -0.29, p 0.000). There were no statistically significant changes for men in the level of frailty by whether they received the federal or state program.

We find our results in Table 2 were robust, obtaining similar results in magnitude and sign using OLS and PSM methods controlling for covariates that included demographic characteristics and health risk factors for frailty (see Table 3). S2 Fig. shows the density curves of the propensity scores before matching and provides more detailed information about the quality of the matching. Table 3 shows that the frailty index and frailty level differences for women are statistically significant after correcting for multiple hypotheses testing in column HB across the DID estimation methods.

**Table 3:** Difference-in-Differences (DID) of the Means, OLS Regressions, and Propensity Score Matching.

	State-Federal (W2-W1)											
	DID of the means				DID regressions				DID propensity score matching			
	Diff.(SE)	95% CI	*P*	HB	Coef. (SE)	95% CI	*P*	HB	Coef.(SE)	95% CI	*P*	HB
	Men											
Weight loss	-0.01 (0.03)	(-0.06, 0.05)	0.831		0.00 (0.04)	(-0.08, 0.08)	0.985		-0.02 (0.04)	(-0.02, -0.02)	0.320	
Weakness	0.00 (0.03)	(-0.06, 0.06)	0.915		-0.01 (0.05)	(-0.11, 0.10)	0.914		0.02 (0.05)	(0.01, 0.02)	0.355	
Exhaustion	-0.06 (0.02)	(-0.11, -0.02)	0.002	††	-0.07 (0.03)	(-0.13, -0.01)	0.033		-0.07 (0.04)	(-0.07, -0.07)	0.030	
Slow pace	0.02 (0.03)	(-0.05, 0.08)	0.626		-0.01 (0.05)	(-0.11, 0.09)	0.902		-0.02 (0.05)	(-0.02, -0.01)	0.365	
Low physical activity	-0.07 (0.04)	(-0.16, 0.01)	0.080		-0.09 (0.07)	(-0.21, 0.04)	0.177		-0.06 (0.06)	(-0.06, -0.05)	0.191	
Frailty Index [0 to 5]	-0.14 (0.07)	(-0.28, 0.00)	0.056		-0.18 (0.12)	(-0.41, 0.06)	0.141		-0.16 (0.11)	(-0.17, -0.15)	0.072	
Frailty Level [0 = Not frail, 1 = pre-frail, and 2 = frail]	-0.05 (0.05)	(-0.14, 0.04)	0.285		-0.07 (0.07)	(-0.21, 0.08)	0.358		-0.04 (0.07)	(-0.05, -0.04)	0.256	
	Women											
Weight loss	-0.04 (0.03)	(-0.09, 0.02)	0.157		-0.02 (0.04)	(-0.10, 0.06)	0.586		-0.05 (0.05)	(-0.05, -0.04)	0.169	
Weakness	-0.16 (0.04)	(-0.23, -0.09)	0.000	††	-0.13 (0.06)	(-0.24, -0.02)	0.022		-0.09 (0.06)	(-0.09, -0.08)	0.057	
Exhaustion	-0.10 (0.03)	(-0.15, -0.05)	0.000	††	-0.13 (0.04)	(-0.21, -0.05)	0.003	††	-0.13 (0.04)	(-0.13, -0.12)	0.000	††
Slow pace	-0.08 (0.03)	(-0.15, -0.02)	0.008	††	-0.08 (0.05)	(-0.18, 0.02)	0.117		-0.09 (0.05)	(-0.10, -0.09)	0.049	
Low physical activity	-0.06 (0.04)	(-0.14, 0.03)	0.195		-0.06 (0.06)	(-0.19, 0.07)	0.351		-0.10 (0.07)	(-0.11, -0.09)	0.074	
Frailty Index [0 to 5]	-0.43 (0.07)	(-0.57, -0.29)	0.000	††	-0.42 (0.12)	(-0.65, -0.19)	0.000	††	-0.44 (0.13)	(-0.45, -0.43)	0.000	††
Frailty Level [0 = Not frail, 1 = pre-frail, and 2 = frail]	-0.22 (0.04)	(-0.31, -0.13)	0.000	††	-0.22 (0.07)	(-0.36, -0.08)	0.002	††	-0.25 (0.08)	(-0.26, -0.24)	0.001	††

Notes: Diff. = Difference; Coef. = Coefficient SE = Standard errors

††p < .05 after HB correction.

Regarding potential sample-selection issues, we found that those who died after W1 were on average older and that differences in age between deceased and surviving respondents were similar in both towns. As earlier noted, we found few statistically significant differences in socioeconomic characteristics between the state- and federal-program towns. We also found no evidence of attrition bias, i.e., differences in socioeconomic characteristics between attriters and those remaining in the panel were not statistically significant in either town (see S4 Table for men and S5 Table for women). We also compared age and gender as collected in the listing for not-completed and completed interviews in W1 to assess any potential bias between the listing and W1. S6 Table shows these differences were not statistically significant in either town. We analyzed item non-response per outcome variable and found those who did not complete the grip-strength and walking-speed tests are older than those who did. The earlier findings are similar in the state and federal program towns and the difference of the differences are not statistically significant.

## Discussion

Our results show different effects in frailty status for women in the state and federal-level NCP and no effects for men in either program. Non-contributory pensions may affect frailty through different pathways. The non-contributory pension provides households with additional income, alleviating at least some of their financial strain. Pension recipients decide which fraction of this supplemental income they use for their own consumption or saving and which fraction they contribute to the pool of household resources shared by other household members. The fraction given to the pool of household resources may be used for household consumption that benefits all household members or it may be devoted to the pension recipient or specific family members.

Greater income for older household members may give them more prominence in household decision making—or, alternatively, lead to their abuse. Previous research has found 2.6% of older adults in Mexico City reported abuse or mistreatment [56]. Our survey found the introduction of the NCP may have led to lower levels of abuse. Our survey asked older adults whether they were fearful somebody would take their money; the proportion expressing such a fear decreased from 2.4% in W1 to 0.7% in W2. Similarly, 1.1% of older adults in W1 reported feeling verbally or physically abused, a proportion that decreased to 0.1% in W2. Altogether, our findings do not indicate that the introduction of the NCP increased abuse of its recipients.

The NCP may also affect household income by affecting paid labor of the recipient or private family transfers. Recipients may substitute pension income for work income, and family members may similarly substitute pension income for family transfers. Such changes could offset the impact of the non-contributory pension on recipients’ wellbeing. It is difficult to capture the exact mechanism of resource allocation after pension receipt unless a protocol and instrument is designed to reflect the potential pathways before pension implementation [57]. Unfortunately, this study, like many previous ones, does not have an instrument designed to analyze potential pathways. Most prior studies have analyzed self-reported or more objective measures of wellbeing at the household or individual level and discuss *a posteriori* the potential mechanisms or pathways.

We hypothesize that the effect of the NCP on stabilizing or even reducing the level of frailty would depend on how much pension resources helped improve consistent food intake, health care use, and access to medicines for the recipient. In this context, we note our findings that the monthly state-level pension decreased frailty for women while the federal pension paid every other month increased frailty for women, with neither affecting frailty for men, are consistent with other studies [58,59] showing that frailty is a dynamic process and that individuals progress through different stages as they age.

Our hypothesis suggests only women in the monthly state-level program may be using pension resources to steadily improve food intake, health care use, and access to medicines. Previous research using the same data we do, supports our contention. A previous study [20] found that six months after the introduction of the state-level program there was an increase in health care utilization and access to medicines by older adults and an increase in the proportion of out-of-pocket health expenditures paid by older adults rather than by relatives. It also found improvements in cognition as measured by word recall and respiratory flow and an increase in food availability for households that had reported suffering hunger or lack of enough food to eat. They also found that a substitution of public transfers for private ones, but that this crowding-out effect represented 36.5% of the non-contributory pension, and a 4.5 percentage points decline of work for pay among recipients. In other words, there was evidence of some but not complete crowding out, while some health outcomes and health care use increased sharply.

Another study [21] found that the state-level program paid every month resulted in improvements in health-care use but that the federal program disbursed every two months did not. It also showed the state-level program increased food availability more than the federal-level program did. The state program further smoothed food expenditure as shown by a regular pattern of average food expenditure throughout the month, while households in the federal program showed a more irregular pattern of food expenditure, particularly in the second month following a pension payment, than was evident before the introduction of the pension. This is likely because individuals have trouble saving throughout a two-month period. The federal program also had large effects on ownership of durable goods such as cell phones, suggesting that its more infrequent payments in greater amounts led recipients to buy durable goods and neglect basic needs.

Other studies analyzing the effects of the federal-level pension found 11 months after the introduction of the program recipients had fewer depressive symptoms and increased food intake but no substantial change in body-mass index, and increased food and non-food consumption [18,19]. These other studies did not analyze changes in food intake or household patterns in food and non-food expenditures between pension payments, but mainly average effects for the total sample and some subgroups. Altogether, previous research suggests both state and federal pensions can improve food availability and thereby some dimensions of health, but in the longer-term the state-level program paid every month may have greater effects through its steady improvements in food availability and health care utilization.

The differential effects by gender in the state and federal-level programs require further reflection. Consistent with previous studies in Mexico of cash transfer programs, our results show greater improvements for women than for men [19, 60]. In a study of the impact of the federal-level NCP on the nutritional status of beneficiaries, Salinas-Rodríguez and colleagues [19] found that the impact of the program on energy, macronutrient intake, and intake of proteins and carbohydrates was significantly higher among women, the indigenous population, and those of the lowest socioeconomic status. Berhman and Parker [60] found positive health effects for women from the PROGRESA/Oportunidades CCT (conditional cash transfer) programs. Among possible explanations for these effects are that women, while living longer, have equally extended periods of disability, frailty, and chronic disease [61] and less access to formal sources of income during retirement [2]. Supplemental or more likely first-time income receipt via a NCP may therefore have a greater impact on women than men. Put another way, NCP are more likely for women than men to increase adequacy of food intake, ability to buy medicine, and access to health care services, all of which jointly have a direct impact by improving health, including measures of exhaustion, slow pace, and frailty. This latter may explain why the state-level program reduces frailty for women but not men. In contrast, the federal-level program produces a more irregular pattern of food expenditure and no effects on health care utilization that may cause a negative impact on women because of women’s lack of alternative sources of income.

We explored other potential explanations to our findings. Our robustness tests showed no indication that our findings could be explained by sample selection issues or differences between towns. The differing amounts of the pension—about US$5.90 per month at 2014 PPP more for the state than the federal program—is unlikely to explain our results. While reductions in frailty are likely to take some time to occur, and the timing of our subsequent survey meant the state program had been in effect for 18 months and the federal program for only 12 months, our estimates show an increase in frailty for women in the federal-level program but a decrease for those in the state-level program. Moreover, previous studies using our data find no effects on health care utilization but do find an irregular food expenditure pattern for the federal program that may affect frailty. Hence, we consider the frequency of pension payment to be a more important cause of our results than the longer time period the state-level pension had been in effect. That is, our findings demonstrate the importance of payment frequency on longer-term health outcomes.

### Limitations

There are some limitations of our survey that narrow the conclusions we may draw from it. The survey does not include a food diary or more detailed information on household food expenditure needed to assess changes on nutrition. The survey is similar to the HRS, including general questions on household food expenditure at home and out of home during the previous week. It also includes a food availability scale analyzed in previous studies. Nevertheless, the survey does not provide an accurate picture of changes on nutritional status. Further consideration should also be given to the relation of functional ability with other conditions such as depressive symptomatology. For example, a recent study using longitudinal data from England found gait speed inversely associated with depressive symptoms [62]. That is, persons with slower gait speed were more likely to report elevated depressive symptoms, and those who reported sub-threshold or elevated depressive symptoms had slower gait speed and were more likely to subsequently experience a further decrease in their gait speed and physical performance. Our Frailty Index includes an indicator of depressive symptoms, exhaustion, and functional measures such as gait speed; future studies should explore trajectories of depressive symptoms together with performance measures such as gait speed in order to investigate possible bidirectional associations.

### Implications

As mentioned previously, in 2013 the federal program reduced age eligibility to 65 years. Even with this change, the policy implications of our findings remain valid because frailty is more relevant for the oldest old. They also remain valid given that, as of 2018, the federal program is still disbursed every two months. Our findings for the federal-level program may be consistent with previous research showing increases in mortality under the federal program [63]. Nevertheless, more research is needed to understand the longer-term effects of NCP on health using longer panel data.

Our results as well as earlier research suggest that pension recipients may pool a proportion of their resources for household expenditures [29], and increase their participation in household spending decisions[19]. Further research, however, is needed to understand how these pooled resources are allocated by gender of pension recipient. For now, this study does show how short-term effects of pensions paid at least monthly can lead to long-term improvements for women. This work also provides more evidence of the effects of income on a comprehensive measure that provides a better indicator health and wellbeing of older adults.

Despite the rapid growth in the older population in Mexico, public health and social care services for older adults are still scarce, and there are no formal public long-term care programs to support ill or disabled older Mexicans. The rapid growth in the older population in Mexico is taking place in a context of economic hardship and lack of universal coverage of social security or welfare benefits, with most workers not covered by any pension or retirement plan and with access only to recent NCP and CCT programs. This is increasing pressure on governments to improve health and social care for older persons [2].

The few services that are available are divided among the different institutions that provide them. Health services and users are divided by whether they receive services from social security institutions the Ministry of Health at the federal or local level, and private sector providers. This is a highly fragmented system where institutions offer different services, work independently and parallel to each other, and are financed through different mechanisms [64]. Hence, access to and quantity and quality of care varies greatly.

Most government-provided services for the older population are an extension of poverty-alleviation programs or other social development strategies targeting the disadvantaged conditions in which some older adults live. Examples of these are the conditional cash transfer programs described earlier, NCPs first introduced at local level as the study presented here, and the federal NCP.

The effects of these programs have implications for other settings. First, the results of this study may be extrapolated to other middle-income countries. In Yucatan, the state-level NCP provided a monthly amount of US$58.70 PPP, equivalent to 31% of the minimum wage and 44% of average household income, representing a significant income supplement. Its impact could be equally beneficial in similar socioeconomic and demographic settings, including those in other Latin American countries such as Colombia, where such benefits are US$44 in PPP, and Peru, where they are US$75 in PPP.

Second, our results suggest more frequent payment of non-contributory pensions are more likely to lower the risk of frailty among older women, supporting previous literature noting that increased income, better diet, more health care, and more information about health practices can improve health and wellbeing [60]. Other strategies to support older adults should consider the implication of payment frequencies and how they relate to program goals.

Third, given the female aging pattern in Mexico and the differentiated gender effects of the supplementary income, local and federal governments should explore introducing differentiated policies that target women in old age to alleviate their disadvantaged conditions. Future studies should further investigate the longitudinal pathways through which supplemental income programs work, and the reasons behind their homogenous or differentiated gender effects.

Finally, given the changing health and social care needs of older adults and the clear effects of a supplemental income well beyond expected economic outcomes, such as in older women’s frailty status, health and social development ministries should urgently collaborate on specific strategies that integrate and optimize all exiting efforts for improving the well-being of older women.

## Supporting information

S1 Fig Inequality and development indexes of the state and federal pension programs municipalities and other municipalities. (DOCX)

S2 Fig Density curves propensity score before matching. (DOCX)

S1 Table Characteristics of Valladolid and Motul, Yucatan 2005. (DOCX)

S2 Table Program participation rates of government programs. (DOCX)

S3 Table OLS regressions to test for common trends in households with individuals 70 or older in the state and federal pension programs municipalities, 1990, 1995, 2000, 2005, and 2010. (DOCX)

S4 Table Comparison of baseline descriptive characteristics for all baseline, panel, and deceased respondents. (DOCX)

S5 Table Comparison of baseline descriptive characteristics for all baseline, panel, and deceased respondents. (DOCX)

S6 Table Completed interviews and not completed interviews at baseline. (DOCX)
