# Supplementary material for: Non-contributory pension programs and frailty of older adults: Evidence from Mexico
Source: PLoS One. 2018 Nov 2;13(11):e0206792. doi: 10.1371/journal.pone.0206792 (PMC6214535; doi:10.1371/journal.pone.0206792)
Supplement: S1 Fig — (DOCX) [file pone.0206792.s001.docx]

**S1 Fig. Inequality and Development Indexes of the State and Federal Pension Programs Municipalities and Other Municipalities**

S1.1 Fig. Gini Coefficient

S1.2 Fig. Human Development Index

Notes: The Gini coefficient is a measure of statistical dispersion that represents the income distribution of a particular population. The range of values for this coefficient is from 0 to 1. Values closer to 0 represent more equality in the distribution of income and values closer to 1 represent higher inequality in the income distribution. The Human Development Index (HDI) is a composite statistic of life expectancy, education, and income per capita. The min and max values of the indicator are set in order to set the range of the index from 0 to 1, where 1 would represent the highest observed values for the components of the indicator and 0 would represent subsistence values.

Sources: Consejo Nacional de Evaluacion de la Politica de Desarrollo Social [CONEVAL] for Gini Coefficient and United Nations Development Program and Consejo Nacional de Poblacion [CONAPO] for Human Development Index.
