# Supplementary material for: Non-contributory pension programs and frailty of older adults: Evidence from Mexico
Source: PLoS One. 2018 Nov 2;13(11):e0206792. doi: 10.1371/journal.pone.0206792 (PMC6214535; doi:10.1371/journal.pone.0206792)
Supplement: S2 Fig — (DOCX) [file pone.0206792.s002.docx]

**S2 Fig. Density Curves Propensity Score Before Matching**

| S2.1 Fig. Men |  |  |  |  |  |  |  |  |  | S2.2 Fig. Women |
| --- | --- | --- | --- | --- | --- | --- | --- | --- | --- | --- |

Notes: The propensity score of men was [0.103, 0.929] for the state pension program and [0.063, 0.849] for the federal pension program. The propensity score of women is [0.230, 0.922] for the state pension program and [0.098, 0.923] for the federal pension program. We employ the pscore command to estimate the propensity score in Stata version 14. We checked that the balancing property of the propensity score was satisfied. We checked for the overlapping region of support and impose region of common support. After imposing common support, we dropped 4 observations or 0.8% of the sample for men and no observations for women. We conduct Kernel matching using the command attk in Stata version 14. We employ the kernel function Epanechnikov and the bandwidth is 0.06. The standard errors are estimated using the bootstrap method with 1,000 replications.
