# Supplementary material for: Non-contributory pension programs and frailty of older adults: Evidence from Mexico
Source: PLoS One. 2018 Nov 2;13(11):e0206792. doi: 10.1371/journal.pone.0206792 (PMC6214535; doi:10.1371/journal.pone.0206792)
Supplement: S1 Table — (DOCX) [file pone.0206792.s003.docx]

**S1 Table. Characteristics of Valladolid and Motul, Yucatan 2005**

| Variables | State Program  (Valladolid) | Federal Program  (Motul) |
| --- | --- | --- |
|  | % or Index | % or Index |
| Illiterate population 15 years old or above | 10.9 | 11.2 |
| Households with overcrowding | 42.2 | 43.2 |
| Households without electricity | 2.1 | 2.8 |
| Households with earthen floor | 3.6 | 2.9 |
| Households without refrigerator | 24.9 | 28.3 |
| Poverty index | -1.1 | -0.9 |
| Notes: Estimations at the locality level conducted by the Mexican National Population Council (CONAPO) based on the 2005 Mexican Census, INEGI, Mexico, 2005.  Source: CONAPO (2005). | | |
