# Supplementary material for: Non-contributory pension programs and frailty of older adults: Evidence from Mexico
Source: PLoS One. 2018 Nov 2;13(11):e0206792. doi: 10.1371/journal.pone.0206792 (PMC6214535; doi:10.1371/journal.pone.0206792)
Supplement: S2 Table — (DOCX) [file pone.0206792.s004.docx]

**S2 Table. Program Participation Rates of Government Programs**

|  | State Program (Valladolid) | | Federal Program (Motul) | |
| --- | --- | --- | --- | --- |
| Government Programs |  |  |  |  |
|  | % | % | % | % |
|  | W1 | W2 | W1 | W2 |
| Receives Reconocer (State non-contributory pension program) | 0.0 | 97.7 | 0.0 | 0.0 |
| Receives 70 y Más (Federal non-contributory pension program) | 0.0 | 0.0 | 0.0 | 83.8 |
| Receives Oportunidades | 11.0 | 8.1 | 17.7 | 14.2 |
| Receives PROCAMPO | 0.5 | 0.3 | 1.6 | 0.4 |
| Notes: Oportunidades is a poverty alleviation program for urban and rural households in extreme poverty. PROCAMPO is an income supplement program for agricultural workers. | | | | |
