# Supplementary material for: Non-contributory pension programs and frailty of older adults: Evidence from Mexico
Source: PLoS One. 2018 Nov 2;13(11):e0206792. doi: 10.1371/journal.pone.0206792 (PMC6214535; doi:10.1371/journal.pone.0206792)
Supplement: S3 Table — (DOCX) [file pone.0206792.s005.docx]

| **S3 Table. OLS Regressions to Test for Common Trends in Households with Individuals 70 or older in the State and Federal Pension Programs Municipalities, 1990, 1995, 2000, 2005, and 2010** | | | | | |
| --- | --- | --- | --- | --- | --- |
|  | Live alone | Health Insurance | Work Status | Earthen Floor | Sewer System |
|  |  |  |  |  |  |
| State Program | -0.00 | -0.15** | 0.04 | 0.10 | 0.01 |
|  | (0.05) | (0.06) | (0.06) | (0.07) | (0.02) |
| Interaction terms |  |  |  |  |  |
| State* 1995 | 0.08 |  | 0.13 | 0.27 | -0.01 |
|  | (0.18) |  | (0.23) | (0.21) | (0.02) |
| State* 2000 | -0.05 |  | 0.00 | -0.07 | -0.01 |
|  | (0.07) |  | (0.08) | (0.08) | (0.02) |
| State* 2005 | -0.00 | 0.00 |  | 0.02 | -0.01 |
|  | (0.06) | (0.08) |  | (0.08) | (0.02) |
| State* 2010 | -0.06 | 0.22*** | -0.04 | -0.04 | -0.00 |
|  | (0.06) | (0.08) | (0.07) | (0.07) | (0.02) |
| Year |  |  |  |  |  |
| 1995 | -0.01 |  | 0.22 | -0.11 | -0.01 |
|  | (0.12) |  | (0.18) | (0.13) | (0.01) |
| 2000 | 0.01 |  | 0.13** | -0.16*** | -0.01 |
|  | (0.05) |  | (0.05) | (0.05) | (0.01) |
| 2005 | -0.04 | 0.08 |  | -0.20*** | -0.01 |
|  | (0.05) | (0.06) |  | (0.05) | (0.01) |
| 2010 | 0.07 | 0.11** | 0.05 | -0.20*** | -0.00 |
|  | (0.05) | (0.05) | (0.05) | (0.05) | (0.01) |
| Constant | 0.14*** | 0.54*** | 0.16*** | 0.24*** | 0.01 |
|  | (0.04) | (0.04) | (0.04) | (0.05) | (0.01) |
| No. Observations | 1,183 | 989 | 848 | 1,177 | 1,169 |
| R-squared | 0.01 | 0.05 | 0.03 | 0.09 | 0.01 |
| F (interaction) | 0.36 | - | 0.16 | 1.94 | 0.19 |
| Prob > F (interaction) | 0.78 | - | 0.85 | 0.12 | 0.66 |
| \| Notes: ***, **, and * indicates significance at 1%, 5%, and 10%. In order to test for common or parallel trends between the state and federal pension programs communities, we conducted the following OLS regression: $Y_{it}=\beta_{0}+\beta_{1}P_{i}+\beta_{2}year_{1995}+\beta_{3}year_{2000}+\beta_{4}year_{2005}+\beta_{5}year_{2010}+\beta_{6}\left(P_{i}*year_{1995}\right)+\beta_{7}\left(P_{i}*year_{2000}\right)+\beta_{8}\left(P_{i}*year_{2005}\right)+\beta_{9}\left(P_{i}*year_{2010}\right)+\varepsilon_{it}$   Our outcome variables $Y_{it}$ are: live alone, health insurance, work status, earthen floor, and sewer system. $P_{i}$was a dummy variable for the program$State=1,Federal=0$. $year_{t}$ were year dummies for 1995, 2000, 2006, and 2010. $P_{i}*year_{t}$was the interaction of the program community and year dummy. We used data from the Mexican Census 1990, 1995, 2000, 2005, and 2010. The year 1990 was the reference category for live alone, work status, earthen floor, and sewer system. The year 2000 was the reference category for health insurance. Health Insurance was only available for years 2000, 2005, 2010. Work Status was only available for years 1990, 1995, 2000, and 2010. The coefficients of interest were the interaction terms from 1995 to 2005 before the introduction of the state and federal pension programs. We also conducted a joint F-test of the interaction terms $P_{i}*year_{1995}$, $P_{i}*year_{2000}$, and $P_{i}*year_{2005}$before the introduction of the programs reported in the last two rows of Table C. Joint F-test of interactions $P_{i}*year_{t}$ were not computed for health insurance because this variable was only available in 2000 and 2005 before the introduction of the state and federal pension programs. The F-test showed the coefficients of the interactions terms are not jointly statistically significant in all of the regressions. These results may imply both towns followed similar trends through time.  Source: Census 1990, 1995, 2000, 2005, and 2010 (INEGI). \| \| --- \| | | | | | |
