# Supplementary material for: Non-contributory pension programs and frailty of older adults: Evidence from Mexico
Source: PLoS One. 2018 Nov 2;13(11):e0206792. doi: 10.1371/journal.pone.0206792 (PMC6214535; doi:10.1371/journal.pone.0206792)
Supplement: S4 Table — (DOCX) [file pone.0206792.s006.docx]

| **S4 Table. Comparison of Baseline Descriptive Characteristics for All Baseline, Panel, and Deceased Respondents** | | | | | | |  |
| --- | --- | --- | --- | --- | --- | --- | --- |
|  | State Program | | | Federal Program | | |  |
|  | (Valladolid) | | | (Motul) | | |  |
|  | Men panel 1: Baseline vs. Panel Respondents | | | | | | |
|  | Baseline Respondents | Panel Respondents | Difference | Baseline Respondents | Panel Respondents | Difference | DID |
|  | (a) | (b) | (b) - (a) = (c) | (d) | (e) | (e) - (d) = (f) | (c) - (f) |
| Age | 77.9 | 77.5 | -0.34 | 77.4 | 76.9 | -0.56 | 0.22 |
| Marital status |  |  |  |  |  |  |  |
| Single/Divorced/Separated/Widowed | 0.3 | 0.3 | -0.01 | 0.4 | 0.4 | 0.00 | -0.02 |
| Married or consensual union | 0.7 | 0.7 | 0.01 | 0.6 | 0.6 | 0.00 | 0.02 |
| Mean years of education | 2.6 | 2.4 | -0.21 | 2.5 | 2.3 | -0.20 | 0.00 |
| Live alone | 0.1 | 0.1 | -0.01 | 0.1 | 0.1 | -0.01 | 0.00 |
| Mean no. of household residents | 3.5 | 3.5 | 0.00 | 3.4 | 3.6 | 0.14 | -0.14 |
| No. Observations | 612 | 436 |  | 524 | 357 |  |  |
|  | Men panel 2: Deceased vs Panel Respondents | | | | | | |
|  | Deceased | Panel Respondents | Difference | Deceased | Panel Respondents | Difference | DID |
|  | (a) | (b) | (b) - (a) = (c) | (d) | (e) | (e) - (d) = (f) | (c) - (f) |
| Age | 83.54 | 77.54 | -6.01 *** | 81.73 | 76.85 | -4.88 *** | -1.13 |
| Marital status |  |  |  |  |  |  |  |
| Single/Divorced/Separated/Widowed | 0.37 | 0.26 | -0.11 | 0.48 | 0.38 | -0.11 | 0.00 |
| Married or consensual union | 0.63 | 0.74 | 0.11 | 0.52 | 0.62 | 0.11 | 0.00 |
| Mean years of education | 2.51 | 2.38 | -0.13 | 2.14 | 2.27 | 0.13 | -0.26 |
| Live alone | 0.12 | 0.12 | 0.00 | 0.09 | 0.13 | 0.04 | -0.04 |
| Mean no. of household residents | 3.53 | 3.46 | -0.06 | 3.50 | 3.59 | 0.09 | -0.15 |
| No. Observations | 57 | 436 |  | 56 | 357 |  |  |
| Notes: ***, **, and * indicates significance at 1%, 5%, and 10%. | |  |  |  |  |  |  |
