# Supplementary material for: Non-contributory pension programs and frailty of older adults: Evidence from Mexico
Source: PLoS One. 2018 Nov 2;13(11):e0206792. doi: 10.1371/journal.pone.0206792 (PMC6214535; doi:10.1371/journal.pone.0206792)
Supplement: S5 Table — (DOCX) [file pone.0206792.s007.docx]

| **S5 Table. Comparison of Baseline Descriptive Characteristics for All Baseline, Panel, and Deceased Respondents** | | | | | | |  |
| --- | --- | --- | --- | --- | --- | --- | --- |
|  | State Program | | | Federal Program | | |  |
|  | (Valladolid) | | | (Motul) | | |  |
|  | Women panel 1: Baseline vs. Panel Respondents | | | | | | |
|  | Baseline Respondents | Panel Respondents | Difference | Baseline Respondents | Panel Respondents | Difference | DID |
|  | (a) | (b) | (b) - (a) = (c) | (d) | (e) | (e) - (d) = (f) | (c) - (f) |
| Age | 77.69 | 77.06 | -0.63 * | 77.26 | 76.89 | -0.37 | -0.26 |
| Marital status |  |  |  |  |  |  |  |
| Single/Divorced/Separated/Widowed | 0.65 | 0.65 | 0.00 | 0.56 | 0.56 | 0.00 | -0.01 |
| Married or consensual union | 0.35 | 0.35 | 0.00 | 0.44 | 0.44 | 0.00 | 0.01 |
| Mean years of education | 1.90 | 1.82 | -0.07 | 2.01 | 1.97 | -0.04 | -0.03 |
| Live alone | 0.13 | 0.13 | 0.01 | 0.12 | 0.14 | 0.01 | -0.01 |
| Mean no. of household residents | 3.40 | 3.31 | -0.09 | 3.55 | 3.54 | -0.02 | -0.07 |
| No. Observations | 710 | 522 |  | 505 | 332 |  |  |
|  | Women panel 2: Deceased vs Panel Respondents | | | | | | |
|  | Deceased | Panel Respondents | Difference | Deceased | Panel Respondents | Difference | DID |
|  | (a) | (b) | (b) - (a) = (c) | (d) | (e) | (e) - (d) = (f) | (c) - (f) |
| Age | 83.10 | 77.06 | -6.04 *** | 80.36 | 76.89 | -3.47 *** | -2.57 ** |
| Marital status |  |  |  |  |  |  |  |
| Single/Divorced/Separated/Widowed | 0.77 | 0.65 | -0.12 ** | 0.58 | 0.56 | -0.02 | -0.11 |
| Married or consensual union | 0.23 | 0.35 | 0.12 ** | 0.42 | 0.44 | 0.02 | 0.11 |
| Mean years of education | 1.72 | 1.82 | 0.10 | 1.59 | 1.97 | 0.38 | -0.28 |
| Live alone | 0.04 | 0.13 | 0.09 *** | 0.05 | 0.14 | 0.08 ** | 0.01 |
| Mean no. of household residents | 3.90 | 3.31 | -0.59 ** | 4.08 | 3.54 | -0.55 * | -0.04 |
| No. Observations | 69 | 522 |  | 59 | 332 |  |  |
| Notes: ***, **, and * indicates significance at 1%, 5%, and 10%. | |  |  |  |  |  |  |
