# Supplementary material for: Non-contributory pension programs and frailty of older adults: Evidence from Mexico
Source: PLoS One. 2018 Nov 2;13(11):e0206792. doi: 10.1371/journal.pone.0206792 (PMC6214535; doi:10.1371/journal.pone.0206792)
Supplement: S6 Table — (DOCX) [file pone.0206792.s008.docx]

| **S6 Table. Completed Interviews and Not Completed Interviews at Baseline** | | | | |  |  |  |  |  |
| --- | --- | --- | --- | --- | --- | --- | --- | --- | --- |
|  | State Program Not completed % or Mean (SD) | State Program Completed % or Mean (SD) | Difference | *P* |  | Federal Program Not completed % or Mean (SD) | Federal Program Completed % or Mean (SD) | Difference | *P* |
| *Covariates* |  |  |  |  |  |  |  |  |  |
| Age | 77.0 (6.5) | 77.8 (6.6) | 0.8 | 0.339 |  | 77.0 (5.8) | 77.3 (6.5) | 0.3 | 0.784 |
| Gender [1=male, 0=female] | 49.3 | 46.3 | -3.0 | 0.626 |  | 51.7 | 50.9 | -0.8 | 0.934 |
| No. Observations | 71 | 1,322 |  |  |  | 29 | 1,029 |  |  |
| Notes: SD = Standard deviation. Not completed interviews were refusals, death or changed address between listing and baseline survey. | | | | | | | |  |  |
